# Screening of miRNAs as Prognostic Biomarkers for Colon Adenocarcinoma and Biological Function Analysis of Their Target Genes

**DOI:** 10.3389/fonc.2021.560136

**Published:** 2021-03-19

**Authors:** Guoliang Zheng, GuoJun Zhang, Yan Zhao, Zhichao Zheng

**Affiliations:** ^1^ Department of Gastric Surgery, Cancer Hospital of China Medical University (Liaoning Cancer Hospital and Institute), Shenyang, China; ^2^ Department of Pathophysiology, College of Basic Medicine Science, China Medical University, Shenyang, China

**Keywords:** colon adenocarcinoma, The Cancer Genome Atlas (TCGA) database, miRNA, prognosis, target genes

## Abstract

We constructed a prognostic risk model for colon adenocarcinoma (COAD) using microRNAs (miRNAs) as biomarkers. Clinical data of patients with COADs and miRNA-seq data were from TCGA, and the differential expression of miRNAs (carcinoma *vs.* para-carcinoma tissues) was assessed using R software. COAD data were randomly divided into Training and Testing Sets. A linear prognostic risk model was constructed using Cox regression analysis based on the Training Set. Patients were classified as high-risk or low-risk according to the score of the prognostic model. Survival analysis and receiver operating characteristic (ROC) curves were used to evaluate model performance. The gene targets in the prognostic model were identified and their biological functions were analyzed. Analysis of COAD and normal cell lines using qPCR was used to verify the model. There were 134 up-regulated and 140 down-regulated miRNAs. We used the Training Set to develop a prognostic model based on the expression of seven miRNAs. ROC analysis indicated this model had acceptable prediction accuracy (area under the curve=0.784). Kaplan-Meier survival analysis showed that overall survival was worse in the high-risk group. Cox regression analysis showed that the 7-miRNA Risk Score was an independent prognostic factor. The 2,863 predicted target genes were mainly enriched in the MAPK, PI3K-AKT, proteoglycans in cancer, and mTOR signaling pathways. For unknown reasons, expression of these miRNAs in cancerous and normal cells differed somewhat from model predictions. Regardless, the 7-miRNA Risk Score can be used to predict COAD prognosis and may help to guide clinical treatment.

## Introduction

Colon cancer is among the most common malignancies worldwide and is associated with high morbidity and mortality (1). Colon adenocarcinoma (COAD) is the most common type of colon cancer. The potential clinical strategies for treatment of COAD include surgery, chemotherapy, radiotherapy, and targeted therapies. However, because of the incomplete understanding of the pathogenesis of COAD, there are no targeted treatments currently used in clinical practice. Chemotherapy regimens are generally is limited by poor drug bioavailability, multidrug resistance, and high toxicity, and these can lead to significant adverse effects and reduce treatment efficacy (2). Further studies of the pathogenesis and progression of COAD are thus needed so that new treatments can be developed.

High-throughput sequencing and related developments have led researchers to examine the roles of different microRNAs (miRNAs) in different diseases. Initial research on miRNAs reported that they did not encode proteins and suggested that they had no biological function. More recent research showed relationships of the expression of multiple miRNAs with tumor pathogenesis and progression, and with patient prognosis (3–6). The systematic study of specific miRNAs may provide a general understanding of tumorigenesis, help to elucidate the pathogenesis of different human malignancies, and lead to the development of novel tools that can be used for treatment and prediction of prognosis.

In the present study, we developed and validated a prognostic risk model using miRNA-seq data of COAD from The Cancer Genome Atlas (TCGA) database. We then analyzed the functions of genes that were the predicted targets of the miRNAs in the model. Finally, we verified the reliability and effectiveness of the differential expression of miRNAs in the model using qPCR experiments in normal and cancerous cells. The general goals of this study were to determine the relationships of different miRNAs with COAD, and to identify specific miRNAs that may be used as biomarkers to supplement the traditional histopathological prognostic factors and improve the individualized treatment of COAD patients.

## Methods

### miRNA and Clinicopathological Data

COAD transcriptome expression data and clinical data were downloaded from TCGA (https://cancergenome.nih.gov/) and subjected to filtering as follows: *i*) Primary Site: Colon; *ii*) Project: TCGA-COAD; *iii*) Disease Type: adenomas and adenocarcinomas; *iv*) Data Category: transcriptome profiling; and *v*) Data Type: Isoform Expression Quantification. The miRNA data [Release version 16.0 (March 22, 2019)] included 39 paracancer tissues and 398 colon cancer tissues. Clinicopathological data of 385 cases were also downloaded. ActivePerl (version 5.26, 64-bit) scripting language was used for integration and extraction of miRNA expression data and clinical data. R software (version 3.6.1) and specific R packages (described below) were used for data processing and analysis. All data were from TCGA, and no further ethical approval was required. All relevant regulations regarding TCGA data access and patient privacy protection were followed.

### Screening for Differentially Expressed miRNAs

The miRNAs differentially expressed in COAD were screened using the edgeR package in R software. The screening criterion was log_2_(fold change) greater than 1.5 and the cutoff for the false discovery rate (FDR) was 0.05. A volcano map of differentially expressed miRNAs was drawn using the ggplot2 package in R software.

### Establishment and Evaluation of a Prognostic Risk Model

COAD patients in this study were randomly assigned to a Training Set or a Testing Set (1:1 ratio). In the Training Set, hazard ratios (HRs) from univariate Cox regression analysis were used to identify miRNAs significantly associated with overall survival (OS), and all significant miRNAs (P < 0.05) were selected as candidate miRNA biomarkers. The candidate miRNAs were then incorporated into a multivariate Cox regression analysis. The importance score of each miRNAs was calculated by supervised principal component analysis, and important miRNAs were selected using 10-fold cross-validation to construct a prognostic risk model based on miRNA expression as follows:

Prognostic score=(β1×expression of gene−1)+(β2×expression of gene2)+… (βn×expression of gene n)

The miRNA prognostic model from the Training Set was used to calculate prognostic scores of the Testing Set and of all patients with COAD. The Training Set, Testing Set, and all patients with COAD were classified as having high-risk or low-risk, based on the median prognostic score in the Training Set. Kaplan-Meier survival curves were used to verify survival differences between the high-risk and low-risk groups. Time-dependent receiver operating characteristic (ROC) analysis was used to assess the predictive power of the prognostic rm2isk model. Then, the prognostic risk model and clinical parameters were analyzed using univariate and multivariate Cox survival analysis to identify the prognostic value of the independent prognostic model, with the results presented as forest plots. These analyses were performed using the survminer, caret, glmnet, survival, and survival ROC packages in R software.

### Predicting Target Genes in the Prognostic Risk Model and Establishing the miRNA-mRNA Co-Expression Network

Three databases (miRDB, miRTarBase, and TargetScan) were used to predict the target genes. To improve prediction accuracy, each gene classified as a target was listed in at least 2 databases. Venn diagrams were drawn using the VennDiagram package in R software.

### Biological Function Analysis of Target Genes

Gene ontology analysis (GO) was used for annotating genes and gene products. This analysis contains terms in three categories: cellular component, molecular function, and biological process. The results of GO analysis and Kyoto Encyclopedia of Genes and Genomes (KEGG) enrichment analysis were drawn using the BiocManager, clusterProfiler, enrichplot, ggplot2, colorspace, and stringi packages in R software. A P-value below 0.05 was considered significant.

### Cell Culture

Human normal colonic epithelial cells (HCoEpiC) and human colorectal adenocarcinoma cells (HT29, SW480) were routinely cultured in a 5% CO_2_ cell incubator with high glucose DMEM containing 10% fetal bovine serum, penicillin (100 U/ml), and streptomycin (100 μg/ml). The medium was changed every 2 or 3 days. When the cell confluence reached about 90%, passaging was conducted (1:2 ratio). After all cells were in the logarithmic growth stage, they were washed with a PBS buffer solution and collected for subsequent experiments.

### Real-Time Polymerase Chain Reaction and Statistical Analysis

Total cellular RNA was extracted using the Trizol reagent (Invitrogen, USA) and RNAs were reverse-transcribed into cDNAs using the miRNA First Strand cDNA Synthesis Kit (Sangon Biotech, China). All miRNA levels were assessed using the miRNAs qPCR Kit (Sangon Biotech, China) and the ABI7500 system (Applied Biosystems, USA). Small nuclear RNA U6 was used as an internal reference. There were three replicates for each sample, and the cycle threshold (Ct) values were averaged. The relative expression of each miRNA was calculated using the 2^−△△^Ct method. **Supplementary Table 1** lists the primer sequences. Data were proceeded by SPSS 20.0 software. All continuous values are presented as mean ± standard deviation. The P value was considered to be statistically significant at the 5% level and P value < 0.05 was considered statistically significant.

## Results

### Work Flow

**Figure 1:**
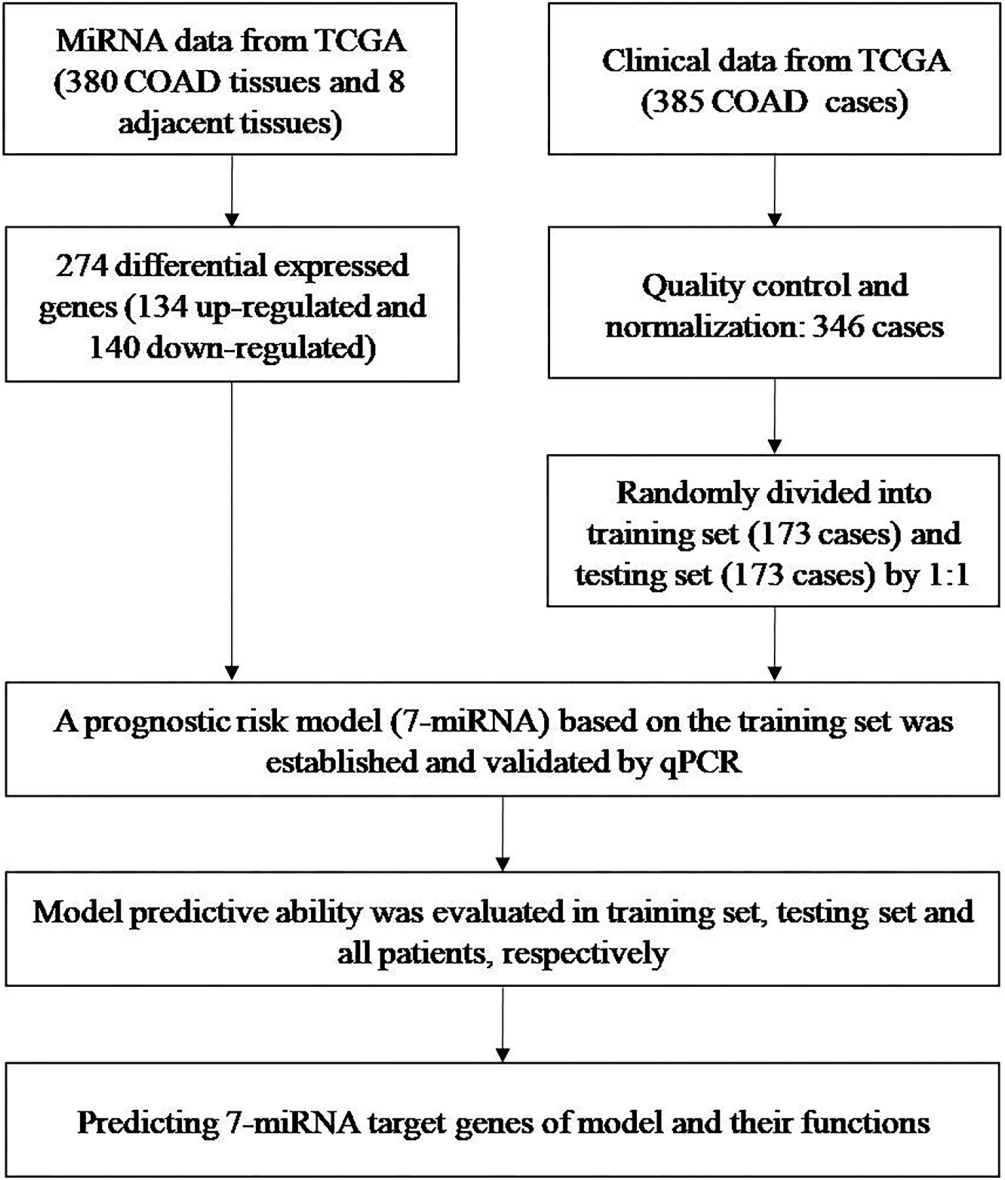
Identification of miRNAs and clinical data from TCGA, development of a predictive model, and predicting the targets of the seven miRNAs in the model.

We examined miRNA data from 380 COAD tissues and eight adjacent normal tissues and clinical data from 385 patients with COADs to develop a prognostic risk model (**Figure 1**). All data were from TCGA.

### miRNA and Clinicopathological Data

We excluded 39 patients whose total survival time was less than 1 month, and enrolled 346 patients with COADs (**Table 1**). There were 189 males and 157 females and the age range was 30–85 years (median: 68 years; mean: 66.35 years). We then randomized these patients into a Training Set (n = 173) and Testing Set (n = 173). These two groups had no significant differences in clinical characteristics (all P > 0.05).

**Table 1:** Demographic and clinical characteristics of COAD patients from TCGA.

Variable	Total (n=346)	Training Set (n=173)	Testing Set (n=173)	χ^2^	*P* value
**Age at diagnosis (years)**	66.35 ± 12.56	66.34 ± 12.16	66.35 ± 12.98	1.091	0.569
**Gender**				0.145	0.746
Male	189	96	93		
Female	157	77	80		
**Stage**				5.991	0.112
I	62	34	28		
II	136	54	77		
III	98	51	47		
IV	50	29	21		
**T Stage**				1.293	0.731
T1	8	4	4		
T2	62	35	27		
T3	240	117	123		
T4	36	17	19		
**Positive nodes** N				0.969	0.325
N0	205	98	107		
N+	141	75	66		
**Distant metastasis**				1.496	0.222
M0	296	144	152		
M1	50	29	21		

### Screening for Differentially Expressed miRNAs

**Figure 2:**
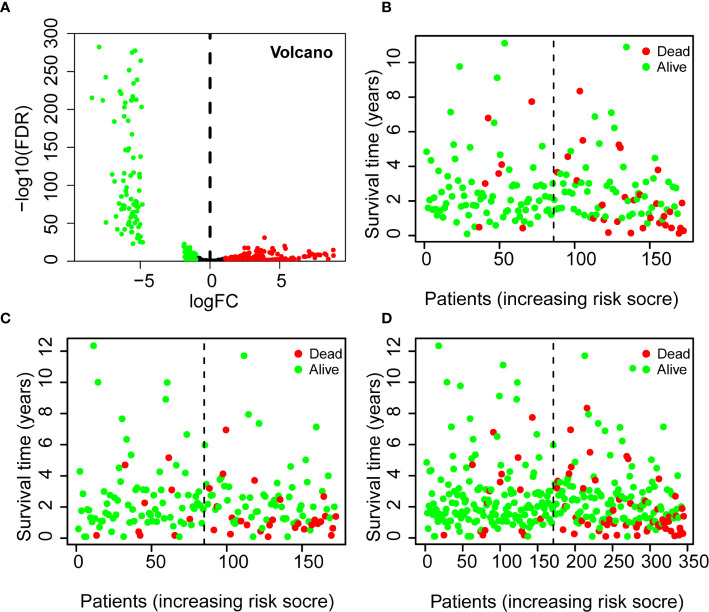
Differential expression of miRNAs in patients with COADs (volcano plot) **(A)** and Using of the 7-miRNA Risk Score to predict survival status and survival time in the Training Set **(B)**, Testing Set **(C)**, and all patients **(D)**. A Red: up-regulated miRNAs [log_2_(FC) > 1.5, P < 0.05]; Green: down-regulated miRNAs [log_2_(FC) < 1.5, P < 0.05]. B–D Green: alive; red: dead; dotted vertical line: risk score curve separating the low-risk and high-risk groups. FC, fold-change.

Analysis of the miRNA data from COAD tissues (n = 380) and paracancer tissues (n = 8) indicated there were 274 differentially expressed miRNAs (**Figure 2A**). There were 134 miRNAs (48.9%) with significant up-regulation and 140 miRNAs (51.1%) with significant down-regulation.

### Establishment and Evaluation of Prognostic Risk Models

Multivariate Cox regression analysis of the Training Set led to the following prognostic risk model based on the expression of seven miRNAs (**Table 2**):

$$\begin{array}{l} \text{Prognostic}\;\text{risk}\;\text{score}=(-0.655\times \text{miR}-194-3\text{p})+(-0.798\times \text{miR}-21-3p)\\ +(-0.429\times \text{miR}-3677-3\text{p})+(-0.381\times \text{miR}-125\text{b}-5\text{p})+(0.441\times \text{miR}-193\text{b}-5\text{p})\\ +(0.858\times \text{miR}-3648)+(0.431\times \text{miR}-193\text{a}-5\text{p}) \end{array}$$

**Table 2:** Parameters in the prognostic risk score* for COAD based on the expression of seven miRNAs.

miRNA	Coef	HR	95% CI		*P* value
miR-194-3p	−0.655	0.519	0.339−0.795		**0.003**
miR-21-3p	−0.798	0.451	0.242−0.837		**0.012**
miR-3677-3p	−0.429	0.651	0.462−0.918		**0.014**
miR-125b-5p	−0.381	0.683	0.451−1.034		0.071
miR-193b-5p	0.441	1.554	0.97−2.478		0.064
miR-193a-5p	0.431	1.538	1.008−2.346		**0.046**
miR-3648	0.858	2.359	1.441−3.862		**0.001**

CI, confidence interval; Coef, regression coefficient; HR, hazard ratio.

*Prognostic risk score = (−0.655 × miR-194-3p) + (−0.798 × miR-21-3p) + (−0.429 × miR-3677-3p) + (−0.381 × miR-125b-5p) + (0.441 × miR-193b-5p) + (0.431× miR-193a-5p) + (0.858 × miR-3648). Values in bold indicate P less than 0.05.

Thus, three miRNAs (miR-193b-5p, miR-193a-5p, miR-3648) were associated with increased risk of death, and four miRNAs (miR-194-3p, miR-21-3p, miR-3677-3p, miR-125b-5p) were associated with reduced risk of death. Analyses of the Training Set, Testing Set, and all patients indicated that each high-risk group had a greater mortality rate than each low-risk group (**Figures 2B–D**).

**Figure 3:**
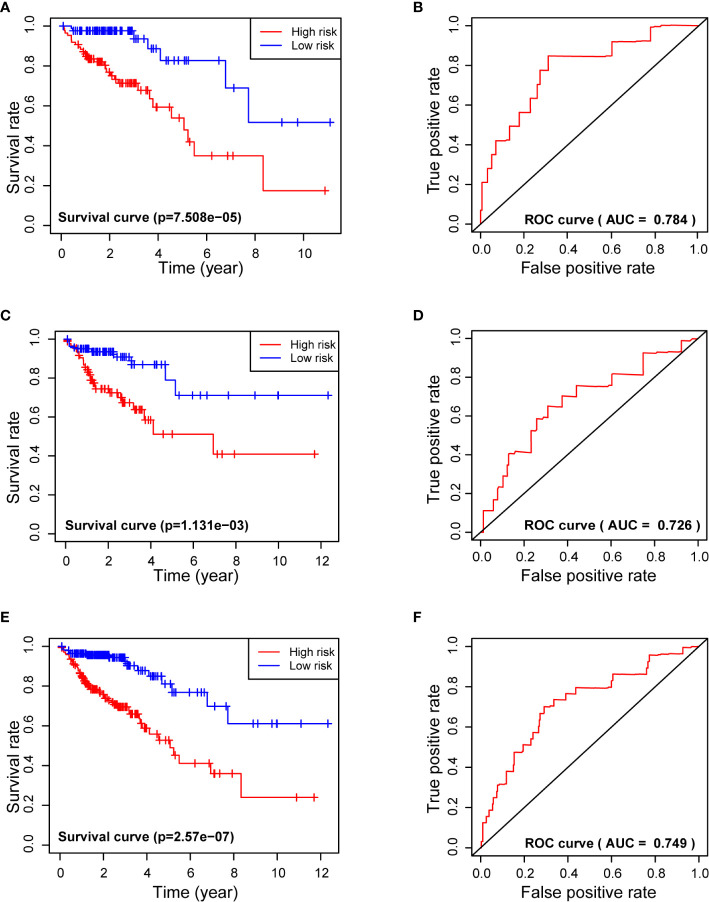
Use of the 7-miRNA Risk Score for analysis of survival times (left) and receiver operating characteristic analysis (right) of the Training Set **(A, B)**, Testing Set **(C, D)**, and all patients **(E, F)**. “+” represents censored data in **(A, C, E)**.

Kaplan-Meier analysis of the Training Set showed that the 3-year survival rate of the high-risk group was significantly lower than that of the low-risk group (P = 7.508×10^−5^). Analysis of the Test Set and of all patients led to similar results. These results thus suggest that the 7-miRNA Risk Score can be used to predict 3-year survival in patients with COADs. Moreover, the ROC curves of the Training Set, Test Set, and all COAD patients were relatively smooth, and the area under the curve (AUC) for each dataset was greater than 0.7, indicating the model had an acceptable prognostic performance (**Figures 3A–F**).

**Figure 4:**
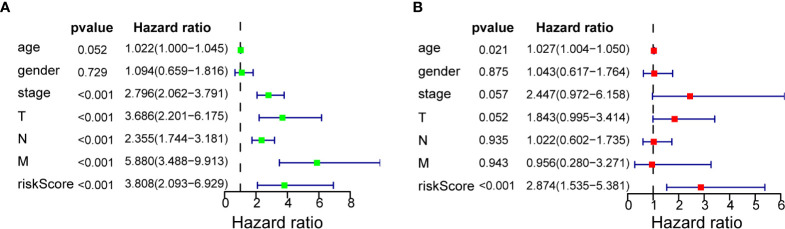
Cox regression analysis of 7-miRNA Risk Score for overall survival in COAD. **(A)** Univariate analysis. **(B)** Multivariate analysis.

Univariate Cox regression analysis (**Figure 4A**) showed that TNM stage, T stage, N stage, metastasis, and 7-miRNA Risk Score were significantly correlated with prognosis (P < 0.05), but age and gender had no effect. Multivariate Cox regression analysis indicated that the 7-miRNA Risk Score was an independent prognostic factor (HR = 2.874, P < 0.001), but none of the other examined variables had significant effects (**Figure 4B**).

### Identification of miRNA–mRNA Regulatory Network

**Figure 5:**
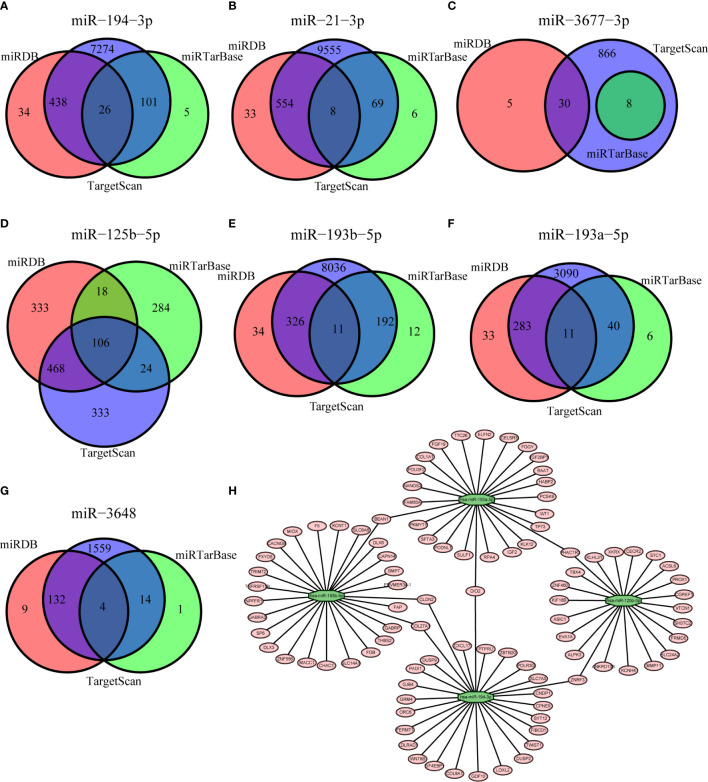
Prediction of genes targeted by each of the miRNAs based on the miRDB (red), miRTarBase (green), and TargetScan (blue) databases (**A–G**, Venn diagrams) and the resulting miRNA-mRNA regulatory network **(H)**.

We used three different databases (miRDB, miRTarBase, and TargetScan) to predict the genes targeted by each of the seven miRNAs in our model (**Figures 5A–G**). The Venn diagram for each miRNA shows the number of target genes from each database and number target genes that any two or three databases had in common. These results led to the identification of a miRNA-mRNAs regulatory network that shows the relationships of these seven miRNAs and their target genes (**Figure 5H**).

### Gene Ontology Analysis and Kyoto Encyclopedia of Genes and Genomes Enrichment Analysis

**Figure 6:**
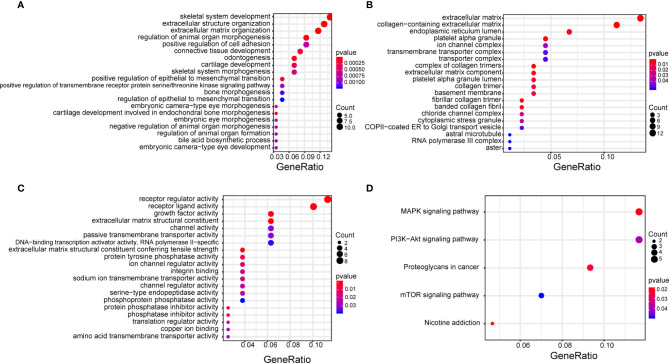
GO enrichment analysis of target genes that function in biological processes **(A)**, cell composition **(B)**, molecular function **(C)**, and KEGG pathway enrichment analysis **(D)**. The size of each circle correlates with the number of genes in the pathway, and color depth represents the −log_10_(FDR) value. GO, Gene Ontology; KEGG, Kyoto Encyclopedia of Genes and Genomes.

GO and KEGG enrichment analysis showed that the target genes were mainly enriched in the MAPK signaling pathway, PI3K-AKT signaling pathway, proteoglycans in cancer signaling pathway, and the mTOR signaling pathway (**Figures 6A–D**).

### qPCR Verification of Results

**Figure 7:**
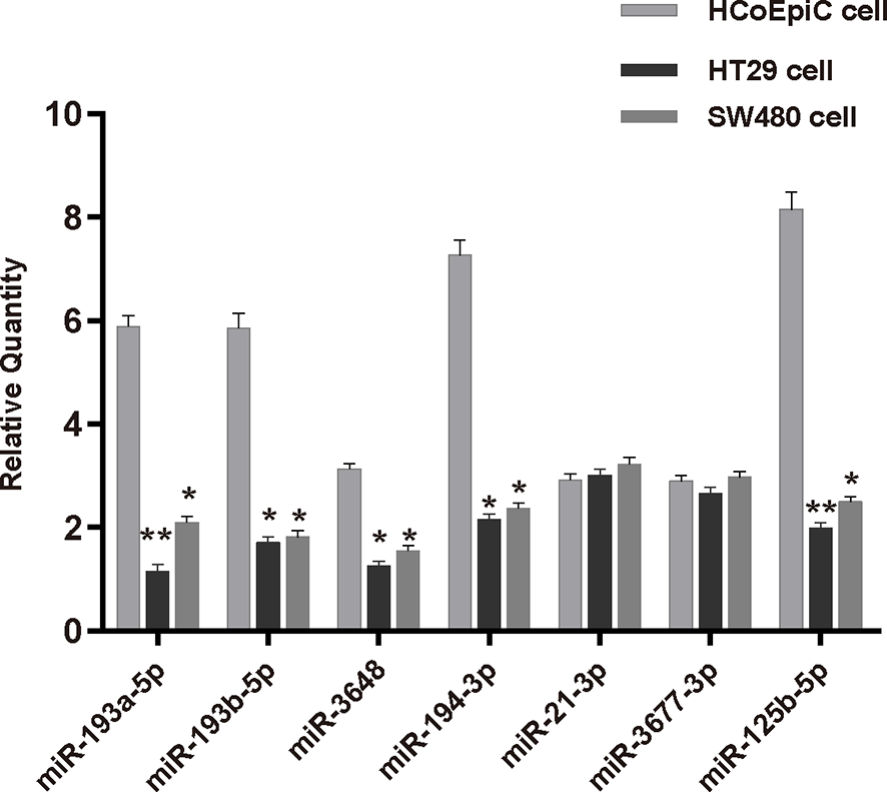
Expression of seven miRNAs from the prognostic model in human normal colonic epithelial cells (HCoEpiC) and human colorectal adenocarcinoma cells (HT29, SW-480). *p < 0.05; **p < 0.01.

We verified the results of our model by performing qPCR analysis in HCoEpiC cells, HT29, and SW480 cells (**Table 3** and **Figure 7**). These results showed that five of the seven miRNAs—miR-193a-5p, miR-193b-5p, miR-3648, miR-194-3p, and miR-125b-5p—were significantly down-regulated in COAD cells and that two of the miRNAs—miR-21-3p and miR3677-3p—had no significant difference in expression between the two cell lines.

**Table 3:** Relative expression of seven miRNAs from the prognostic model in normal colon cells (HCoEpiC), colon cancer cells (HT29, SW-480).

Name	HCoEpiC cell	HT29 cell	SW - 480 cell	P1 value	P2 value
miR-193a-5p	5.886 ± 0.206	1.158 ± 0.126	2.096 ± 0.119	<0.01	<0.05
miR-193b-5p	5.858 ± 0.289	1.701 ± 0.122	1.813 ± 0.124	<0.05	<0.05
miR-3648	3.132 ± 0.103	1.253 ± 0.096	1.549 ± 0.098	<0.05	<0.05
miR-194-3p	7.268 ± 0.295	2.151 ± 0.101	2.369 ± 0.114	<0.05	<0.05
miR-21-3p	2.921 ± 0.106	3.011 ± 0.114	3.223 ± 0.129	0.373	0.289
miR-3677-3p	2.892 ± 0.114	2.658 ± 0.109	2.968 ± 0.118	0.062	0.186
miR-125b-5p	8.153 ± 0.331	1.986 ± 0.099	2.495 ± 0.103	<0.01	<0.05

P1 = HCoEpiC vs. HT29 cell; P2 = HCoEpiC vs. SW-480 cell.

## Discussion

In this study, we identified differentially expressed miRNAs in COAD by screening TCGA using bioinformatics technology, and we then integrated the expression data with clinical parameters of patients to establish a prognostic risk model, the 7-miRNA Risk Score. The model showed good prognostic performance in the Training Set, Testing set, and in both sets together, and ROC analysis of 3-year overall survival indicated that each dataset had AUC values greater than 0.7. Importantly, our multivariate Cox regression analysis demonstrated that the 7-miRNA Risk Score was significantly and independently associated with prognosis. Our analysis predicted that the target genes and their pathways were mostly related to cancer: MAPK signaling (7), PI3K-AKT signaling (8), and mTOR signaling (9). All of these pathways have well-established roles in the development and progression of gastric cancers. Our results thus indicated that the 7-miRNA Risk Score has potential use for prediction of molecular pathogenesis, clinical progression, and prognosis in patients with COADs. Clinical studies are needed to establish whether the 7-miRNA Risk Score can help in the early diagnosis and individualization of treatments in patients with COADs.

Three of the seven miRNAs in our prognostic model—miR-193a-5p, miR-193b-5p, and miR-3648—were associated with high risk, in that high expression correlated with poor with OS. In agreement with TCGA data, miR-193a-5p, miR-193b-5p, and miR-3648 expression were greater in normal cells than COAD cancerous cells. Interestingly, there have been inconsistent results regarding the effects of mir-193a-5p in different cancers. In particular, Zhang et al. (10) reported that downregulation of mir-193a-5p was associated with lymph node metastasis and poor prognosis in colorectal cancer. However, *in vitro* and *in vivo* studies of pancreatic cancer reported that overexpression of miR-193a-5p contributed to metastasis (11). Only one study provided an extensive examination of mir-193b-5p, and the results suggested that this miRNA may function as a tumor-suppressor by targeting CD44v6 in breast cancer (12). A previous study of miRNA-3648 reported that upregulation of this miRNA led to inhibition of TCF21, and thereby promoted the invasion and metastasis of bladder cancer (13). However, there are no previous reports of the roles of miR-193b-5p and miR-3648 in COAD.

Our TCGA analysis indicated that four of the seven miRNAs were associated with reduced risk: miR-194-3p, miR-21-3p, miR-36 77-3p, miR-125b-5p. Previous studies found that miR-194 functioned as a tumor suppressor. For example, Xia et al. (14) reported that miR-194-3p reversed the effect of SLC12A5 and ZFHX4 in promoting the proliferation, invasion, and metastasis of lung adenocarcinoma *in vitro* and *in vivo* based on their analysis of twist1-centric ceRNA networks. Abuduaini et al. (15) found that miR-194-3p inhibited the metastatic biological behaviors of spinal osteosarcoma cells by repression of MMP-9. There are different reports on the effects of miR-21-3p and mir-125b-5p in cancer. Some research (16) suggested that miR-21-3p suppressed growth and induced apoptosis in HepG2 cells by targeting MAT2A and MAT2B, but other research (17) found that suppression of miR-21-3p enhanced TRAIL-mediated apoptosis in liver cancer stem cells by suppressing the PI3K/Akt/Bad cascade *via* regulation of PTEN. Another study found that inhibition of miR-21-3p led to significantly decreased proliferation and invasion of ovarian and prostate cancer cells (18). There is evidence that miR-3677 is negatively associated with OS (19), but the specific mechanism remains unknown. Currently, there is very little known about the specific function and mechanism of these four miRNAs. Further studies of their relationships with COAD are necessary, as are in-depth studies of their biological functions and potential mechanisms.

Our ROC analysis indicated the AUC value of our model was between 0.7 and 0.8 in the Training Set, Testing Set, and in both datasets together. Notably, the results of our univariate and multivariate Cox regression analysis showed that the 7-miRNA Risk Score was significantly and independently associated with OS (HR = 2.874, P < 0.001).

Yin et al. (20) identified eight key miRNAs closely related to colorectal tumorigenesis by examination of eight NATs, seven colorectal adenomas, and 15 colorectal cancer tissues. However, they did not demonstrate the prognostic potential of these miRNAs as biomarkers for colorectal cancer. Moreover, their sample size was small, so the results may be biased. In contrast, we used 346 COAD samples from TCGA to develop a prognostic model that had satisfactory accuracy for predicting the prognosis of COAD patients.

However, there are some shortcomings in this study. First, the data that were randomly assigned to the Training Set and Testing Set were from a single database. Ideally, a separate set of data should be used as an external Testing Set. Second, the follow-up time of COAD patients was relatively short (average: 30.4 months) and the rate of loss to follow-up was relatively high, and this may affect the reliability of the survival analyses. Thus, it is necessary to recruit more COAD patients and conduct long-term follow-up studies to verify the results presented here. In addition, the complex effects and specific mechanisms of the seven miRNAs identified here need further study. Data from other cohort studies may be useful in validating our conclusions. We are currently collecting clinical specimens and additional data to further evaluate the predictive efficiency of the 7-mRNA Risk Score and its potential clinical utilization.

## Conclusion

We used TCGA to develop a prognostic model for COAD based on the expression of seven miRNAs. ROC analysis indicated this model provided satisfactory performance for the Training Set, Testing Set, and both sets together. The results of our qPCR experiments, which compared the expression of these seven miRNAs in normal and cancerous cell lines, were somewhat contrary to model predictions for reasons yet to be determined. Regardless, our 7-miRNA Risk Score has the potential to predict prognosis in patients with COADs. This model identified a relationship between the expression of seven different miRNAs with COAD, and the results suggest that specific miRNAs may be used as biomarkers to supplement the traditional histopathological prognostic factors. More generally, our results suggest the 7-miRNA Risk Score may be used to predict treatment response, potentially enabling more precise and personalized treatments for COAD patients in the future.

## Data Availability Statement

The datasets presented in this study can be found in online repositories. The names of the repository/repositories and accession number(s) can be found in the article/**Supplementary Material**.

## Author Contributions

GZ made substantial contributions to the conception and design of the study. GJZ contributed to the data acquisition, and data analysis, and interpretation. YZ and ZZ contributed to the drafting of the article or critically revising it for important intellectual content. All authors agreed to be accountable for all aspects of the work in ensuring that questions related to the accuracy or integrity of the work are appropriately investigated and resolved. All authors contributed to the article and approved the submitted version.

## Conflict of Interest

The authors declare that the research was conducted in the absence of any commercial or financial relationships that could be construed as a potential conflict of interest.
