# Supplementary material for: Screening of miRNAs as Prognostic Biomarkers for Colon Adenocarcinoma and Biological Function Analysis of Their Target Genes
Source: Front Oncol. 2021 Mar 19;11:560136. doi: 10.3389/fonc.2021.560136 (PMC8017316; doi:10.3389/fonc.2021.560136)
Supplement: Supplementary file 1 [file Table_1.docx]

**Table S1. Primers used for qPCR.**

| **Gene** | **Forward (5’-3’)** | **Reverse (5’-3’)** |
| --- | --- | --- |
| miR-194-3p | ACACTCCCAGUGGGGCUG | CAGAUAACAGTTGAGAGTACAT |
| miR-21-3p | CTCAACTGGTGTCGTGGAGTCGGCAATTCAGTTGAGACAGCCC | ACACTCCAGCTGGGCAACACCAGTCGATGGGC |
| miR-3677-3p | CAGTGGCCAGAGCCCTGCA | GAACATGTCTGCGTATCTC |
| miR-125b-5p | ACTGATAAATCCCTGAGACCCTAAC | TATGGTTTTGACGACTGTGTGAT |
| miR-193a-5p | CTCAACTGGTGTCGTGGAGTCGGCAATTCAGTTGAGTCATCTCG | CGGCGGTGGGTCTTTGCGGGCG |
| miR-193b-5p | ACACTCCAGCTGGGCGGGGTTTTGAGGGCG | CTCAACTGGTGTCGTGGAGTCGG |
| miR-3648 | GCGAGCACAGAATTAATACGAC | AGCCGCGGGGATCGCCGAGGG |
